# Prophilactic aortic-root reconstruction in Marfan syndrome

**DOI:** 10.1186/1749-8090-8-S1-O41

**Published:** 2013-09-11

**Authors:** Z Szabolcs, E Bartha, B Ágg, K Benke, M Pólos

**Affiliations:** 1 Heart and Vascular Center, Semmelweis University, Budapest, Hungary

## Background

The type “A” aortic dissection (AAD) is a dangerous acute complication of the Marfan syndrome. Not only the native outcome of the AAD is life threatening, but even the surgical treatment of this complication is risky. Despite the improving surgical experience and the more effective cerebral and myocardial protection the lethality of the surgical treatment of AAD at Marfan patients is still high as 10-15 percent. Out of this reasons all the possible medical efforts should be taken to prevent the occurrence of this complication.

## Methods and results

The Hungarian Marfan Foundation was founded just ten-year ago in 2002. During the past decade more than 300 patients were registered in the national marfan registry running by the Foundation. Each registered patient undergoes once ore twice a year an ECHO screening up to the size of the sinus Valsalva (SV). To prevent the occurrence of AAD we operate on prophylactic the patients if the diameter of the SV exceeds the 40 mm and at least one member of the patient’s family has already suffered from AAD. But all Marfan patients without exception has to face surgical intervention if the diameter of SV exceeds the 50 mm. For reconstruction of the aortic-root the Bentall procedure was used at the beginning, but in the last two years the intact aortic valves were routinely preserved by Tirone David procedures. During the last ten-year 90 Marfan patients underwent aortic root reconstruction and 30 of these were operated on prophylactic base.

## Conclusion

There was no hospital lethality in the prophylactic group, and the Kaplan Mayer survival examination has proved the five year survival rate to be as high as 93%. In the light of these excellent results is even more contrasted the 49% five year survival rate in the AAD group, not mentioned it’s 21% hospital lethality. The only conclusion might be taken out of these results: the prophylactic aortic root reconstruction is a safe and useful method, connected with excellent long term survival to prevent the occurrence of AAD.

